# Alzheimer’s disease and microorganisms: the non-coding RNAs crosstalk

**DOI:** 10.3389/fncel.2023.1256100

**Published:** 2024-01-05

**Authors:** Hanieh Mohammadi-Pilehdarboni, Mohammad Shenagari, Farahnaz Joukar, Hamed Naziri, Fariborz Mansour-Ghanaei

**Affiliations:** ^1^Faculty of Medicine and Dentistry and the School of Biological and Behavioural Sciences, Queen Mary University of London, London, United Kingdom; ^2^Gastrointestinal and Liver Diseases Research Center, Guilan University of Medical Sciences, Rasht, Iran; ^3^Department of Microbiology, School of Medicine, Guilan University of Medical Sciences, Rasht, Iran; ^4^Department of Neurology, Thomas Jefferson University, Philadelphia, PA, United States

**Keywords:** Alzheimer’s disease, non-coding RNAs, gut microbiome, viral disease, gut–brain axis, neurodegenerative disorders

## Abstract

Alzheimer’s disease (AD) is a complex, multifactorial disorder, influenced by a multitude of variables ranging from genetic factors, age, and head injuries to vascular diseases, infections, and various other environmental and demographic determinants. Among the environmental factors, the role of the microbiome in the genesis of neurodegenerative disorders (NDs) is gaining increased recognition. This paradigm shift is substantiated by an extensive body of scientific literature, which underscores the significant contributions of microorganisms, encompassing viruses and gut-derived bacteria, to the pathogenesis of AD. The mechanism by which microbial infection exerts its influence on AD hinges primarily on inflammation. Neuroinflammation, activated in response to microbial infections, acts as a defense mechanism for the brain but can inadvertently lead to unexpected neuropathological perturbations, ultimately contributing to NDs. Given the ongoing uncertainty surrounding the genetic factors underpinning ND, comprehensive investigations into environmental factors, particularly the microbiome and viral agents, are imperative. Recent advances in neuroscientific research have unveiled the pivotal role of non-coding RNAs (ncRNAs) in orchestrating various pathways integral to neurodegenerative pathologies. While the upstream regulators governing the pathological manifestations of microorganisms remain elusive, an in-depth exploration of the nuanced role of ncRNAs holds promise for the development of prospective therapeutic interventions. This review aims to elucidate the pivotal role of ncRNAs as master modulators in the realm of neurodegenerative conditions, with a specific focus on Alzheimer’s disease.

## 1 Introduction

Alzheimer’s disease (AD) represents a devastating and globally prevalent neurodegenerative disorder, characterized as the most common form of dementia. Recent statistics from the Centers for Disease Control and Prevention (CDC) reveal that nearly 6 million individuals in the United States alone are afflicted by AD (Dorszewska et al., 2016). The etiology of AD remains enigmatic due to its intricate interplay with various organ systems and multicellular interactions, often initiated by microbial infections. Infections targeting the central nervous system (CNS) present a grave and frequently fatal health concern, with survivors often grappling with enduring neurological impairments (Pluta et al., 2020). To unravel the intricate mechanisms governing the impact of microbes on CNS function, it becomes paramount to delve into the regulatory role of non-coding RNAs (ncRNAs) as their significance in numerous disease states has garnered increasing recognition. Notably, the precise mechanisms underlying the contribution of human viral infections or perturbations in the host microbiome to the onset of AD remain elusive, potentially involving intricate ncRNA-mediated processes. Perturbations in ncRNAs can exert profound effects on disease states, thus emphasizing the critical importance of comprehending these molecules and the factors influencing them. Such insights hold promise for manipulating the mechanisms that influence brain function, offering prospects for ameliorating or delaying the progression of neurodegenerative pathology (Li et al., 2021).

## 2 Clinical and cellular mechanisms in Alzheimer’s disease

Alzheimer’s disease (AD) is characterized by a spectrum of clinical and pathological features, encompassing synaptic loss, neuronal damage, alterations in plasticity, and the aggregation of senile plaques composed of Aβ and tau proteins, ultimately culminating in progressive neural system dysfunction over time (Valenza and Scuderi, 2022). Predominantly, AD patients grapple with memory loss as the cardinal clinical manifestation (Barichello et al., 2013). Additionally, AD patients often exhibit mild language impairments and judgment deficits (Miguel et al., 2020). Neuropsychiatric symptoms, including behavioral changes, can also be observed in individuals with AD (Lyketsos et al., 2011). The pathophysiological landscape of AD is multifaceted, underpinned by various hypotheses, including the roles of (a) amyloid-β, (b) Tau pathology leading to neurofibrillary tangles, (c) mitochondrial dysfunction, and reactive oxygen species (ROS) generation, among others (Alqahtani et al., 2023). Amyloidosis, a hallmark feature of Alzheimer’s disease (AD), is characterized by the aggregation of amyloid peptides within cells and tissues, giving rise to disruptive plaques. Aβ peptides can further self-aggregate through complex mechanisms, leading to organ damage. A central player in the amyloidogenic cascade is the beta-site amyloid precursor protein cleaving enzyme 1 (BACE1) enzyme. It cleaves the amyloid precursor protein (APP) at the β site, leading to the production of amyloid β-1 (Sayad et al., 2022). This event is pivotal in AD as the excessive accumulation of amyloid plaques underlies disease pathogenesis. Intriguingly, alterations in BACE1 expression have been linked to neuropathological conditions, suggesting a tightly regulated process governed by non-coding RNAs (ncRNAs; Zhou et al., 2021; Hao et al., 2022; Sayad et al., 2022).

**Figure 1:**
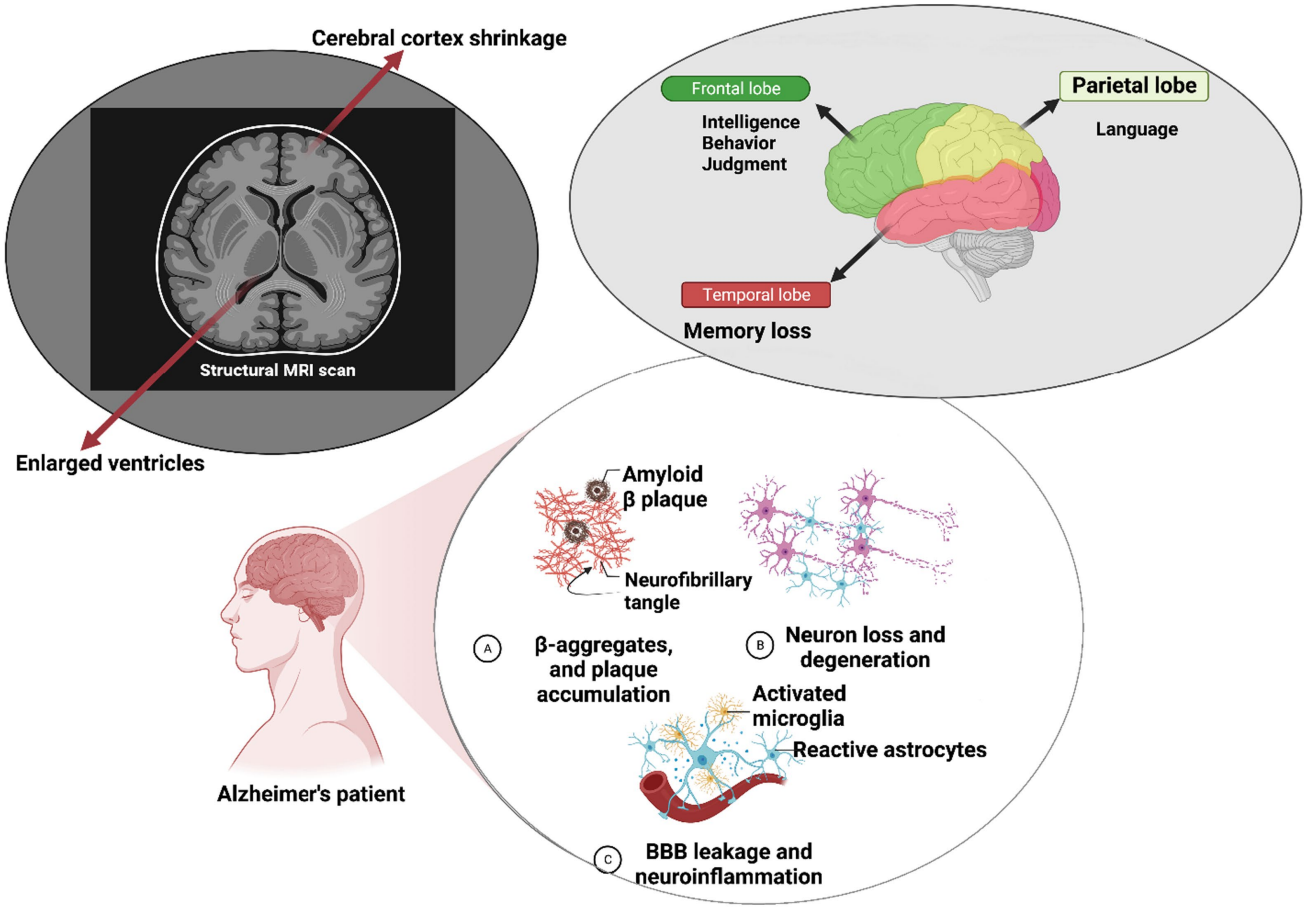
Molecular mechanisms underlying Alzheimer’s disease pathology. At the molecular level within neurons, Alzheimer’s disease (AD) is characterized by the self-aggregation of amyloid-β peptides into plaques and hyperphosphorylation of the tau protein. These pathological events can trigger microglial activation, leading to an increase in blood–brain barrier (BBB) permeability and subsequent neuroinflammation, which results in neural impairments. Clinically, individuals with AD often exhibit a range of symptoms such as deficits in intelligence and judgment behavior, speech difficulties, and memory loss. Brain magnetic resonance imaging (MRI) reveals significant structural changes, including cerebral cortex shrinkage and ventricular enlargement, highlighting the structural alterations associated with the progression of AD.

Neural cells have been reported to produce higher quantities of Aβ compared to other cell types. Aβ is a crucial component involved in intracellular signaling cascades and various physiological functions within the central nervous system (CNS; Bernabeu-Zornoza et al., 2022). Another key aspect of AD is tau pathology. As a microtubule-associated protein, tau contributes to microtubule assembly and related protein interactions. Aberrant tau phosphorylation disrupts microtubule integrity, resulting in synaptic dysfunction and increased neurite branching (Guo et al., 2017). Tau oligomers have been implicated as initiators of deficits in axonal transport, resulting in neural damage and eventual cell death. The mitochondrial damage hypothesis suggests that Aβ accumulation within brain mitochondria can alter their structure and function. These changes are associated with increased Aβ levels, leading to energy generation imbalances and respiratory deficits (Fišar, 2022). Aβ oligomer buildup within the mitochondria can also stimulate bidirectional reactive oxygen species (ROS) production, which can be toxic to neural cells (Araki, 2023). Methylation of mitochondrial tRNA, a small non-coding RNA, has been observed in elderly individuals, whether they are healthy or have Alzheimer’s disease (AD). This methylation can potentially impact mitochondrial protein biosynthesis and contribute to mitochondrial dysfunction, which is an early hallmark of neurodegeneration and age-related disorders (Silzer et al., 2020). Changes in the expressions of tRNA-related fragments (TRFs) have been noted in the hippocampus of AD patients, and these changes appear to be age- and disease-stage dependent (Wu et al., 2021). Gender differences in brain development have significant implications for the investigation of neuropsychiatric and neurodegenerative disorders, including AD. Brain pathology in female patients with AD, compared to male patients, underscores the role of sex differences in the progression of AD (Castro-Aldrete et al., 2023). Aberrant expressions of corticotropin-releasing factor (CRF) in female patients have been correlated with increased tau phosphorylation, a pathological step in the formation of neurofibrillary tangles seen in AD (Bangasser et al., 2017). Gender-associated long non-coding RNAs (lncRNAs), such as SLC25A25-AS1, LY86-AS1, and PWRN1, have been identified as highly dysregulated in AD brains although further studies are warranted to elucidate their precise roles in AD pathology (Cao et al., 2019). Observations through brain MRI reveal ventricle enlargement and cerebral cortex shrinkage, indicative of Alzheimer’s pathology. These structural changes impact memory, intelligence, judgment, behavior, and language in Alzheimer’s patients (Lombardi et al., 2020). Furthermore, disruptions in the blood–brain barrier (BBB) are evident, characterized by altered endothelial cell connections, leading to enhanced fluid transcytosis, pericyte loss, compromised tight junction proteins, and cerebral microhemorrhages (Figure 1; Aragón-González et al., 2022).

## 3 Non-coding RNAs

**Figure 2:**
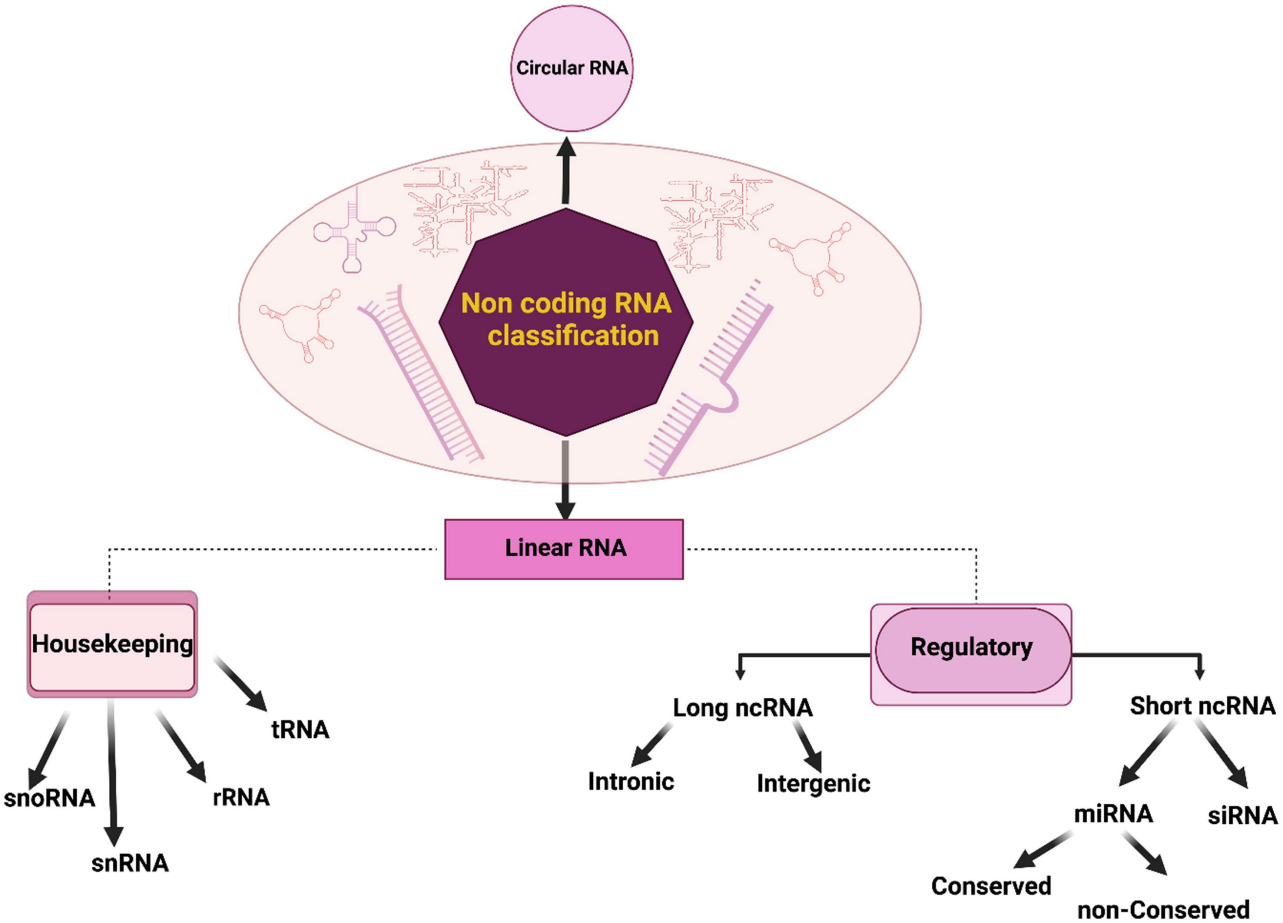
Classification of non-coding RNAs (ncRNAs). In a general categorization, non-coding RNAs (ncRNAs) can be systematically classified into two fundamental groups: circular and linear RNAs. Linear RNAs, further delineated based on their functional roles and expression patterns, segregate into two categories: housekeeping and regulatory elements. The housekeeping group consists of elements that are typically expressed at a constant level, including transfer RNA (tRNA), ribosomal RNA (rRNA), small nuclear RNA (snRNA), and small nucleolar RNA (snoRNA). In contrast, regulatory elements are subdivided into short and long ncRNAs based on their respective lengths. This classification provides a foundational framework for understanding the diverse landscape of ncRNAs and their pivotal roles in cellular processes and disease pathogenesis.

Non-coding RNAs (ncRNAs) constitute a diverse group of RNA molecules that, primarily, do not encode proteins. Their multifaceted roles in cellular processes and pathophysiological conditions have garnered increasing attention in contemporary molecular biology. These ncRNAs participate in gene regulation, epigenetic modifications, and RNA processing, contributing significantly to normal neuronal function (Das et al., 2021). They can be broadly categorized into two major groups: circular RNAs and linear RNAs, as illustrated in Figure 2. Linear RNAs further branch into housekeeping and regulatory elements (Fu, 2014). The housekeeping group encompasses transfer RNA (tRNA), ribosomal RNA (rRNA), small nuclear RNA (snRNA), and small nucleolar RNA (snoRNA). Regulatory elements, based on their lengths, segregate into long ncRNAs (lncRNAs) and short ncRNAs or small ncRNAs (sncRNAs; Zhang et al., 2019). Intergenic and intronic divisions are found within lncRNAs (Ma et al., 2013). Pioneering studies have unveiled that conserved regions within intergenic lncRNAs are significantly enriched in protein-RNA interaction signatures, contrasting non-conserved counterparts such as Malat1, Neat1, and Meg3 (Ruiz-Orera and Albà, 2019). SncRNAs encompass various subsets such as microRNAs (miRNAs), small-interfering RNAs (siRNAs), and piwi-interacting RNAs (piRNAs), among others (Gomes et al., 2013). MiRNAs, highly conserved in evolution, modulate nearly 50% of genes post-transcriptionally, serving as critical regulators in brain development, neuronal processes including apoptosis, proliferation, and differentiation (Gurtan and Sharp, 2013). SiRNAs are involved in posttranscriptional gene silencing although not exclusively (Carthew and Sontheimer, 2009). PiRNAs contribute to diverse neural events, including genomic heterogeneity, neuronal response to injuries, behavior, and memory formation (Kim, 2019; Sato et al., 2023). Regulatory RNAs exhibit the potential to manipulate cellular processes, and their aberrant expressions can underpin disease etiology in specific cases (Statello et al., 2021). In the context of diagnostics, ncRNAs offer valuable tools for molecular biology due to their relative stability in the bloodstream. For example, NEAT1, an ncRNA, plays a pivotal role in chromatin remodeling and microtubule integrity. The downregulation of NEAT1 may enhance the expression of phosphorylated Tau protein, leading to microtubule dysfunction via the Frizzled Class Receptor 3/CSK3β/p-tau pathway (Zhao et al., 2020). Linc00507, another long intergenic non-protein coding RNA, has been associated with AD pathology through enhanced tau phosphorylation (Ni et al., 2022). The dysregulation of NDM29, BC200, 17A, and 51A ncRNAs has been correlated with AD pathology linked to Aβ accumulation (Luo and Chen, 2016). Recent scientific endeavors have spotlighted the hypothesis that microorganisms, particularly those of viral and bacterial origin, may significantly contribute to neurodegenerative processes, potentially employing ncRNAs as mediators in the manifestation of brain impairments.

## 4 Gut microbiome and Alzheimer’s disease

Recent research has shed light on the intricate relationship between the gut microbiome and Alzheimer’s disease (AD) pathology, yet several crucial gaps in understanding remain to be addressed. Human studies, particularly interventional investigations, are needed to draw definitive conclusions regarding the mediating role of non-coding RNAs (ncRNAs) in the initiation and progression of neurodegenerative diseases, including Alzheimer’s, ultimately providing practical avenues for therapeutic interventions (Johnson et al., 2023).

Mounting evidence suggests that dysbiosis of gut microbiota can contribute to AD pathogenesis through microbial metabolites, such as *Bacteroides fragilis* toxins (BFT) and lipopolysaccharide (LPS; Sampson et al., 2016), which actively engage neurotransmitters and ncRNAs in gut–central nervous system (CNS) communication (Huang and Wu, 2021). BFT, for instance, influences the CNS by modulating the NF-κB pathway and upregulating miRNA-146a expression, potentially inciting neuroinflammation and neurodegeneration (Zhao and Lukiw, 2018a). LPS, on the other hand, can increase the expression of miRNAs-34a and 146a, targeting genes implicated in sporadic AD (Sil et al., 2020). While the precise role of miRNAs in gut–brain communication remains elusive, the potential interplay between microbial metabolites and ncRNAs is evident. For instance, butyrate induces miR-375 expression and regulates tryptophan metabolism, a process associated with AD neuropathogenesis and cognitive dysfunction (Moloney et al., 2017).

Exploring the connection between bacterial toxins, gastrointestinal (GI) barrier permeability, and the role of different ncRNAs in facilitating the passage of molecules through the blood–brain barrier (BBB) is essential (Sochocka et al., 2019; Tang et al., 2020). The role of BFT in GI barrier disruption has been documented through synaptic E-cadherin cleavage in GI epithelial cells, emphasizing the need to examine bacterial metabolites with neurotoxic potential (Zhao and Lukiw, 2018b). Additionally, the severity of inflammation in sepsis is closely linked to miR-486-5p expression, a diagnostic biomarker of sepsis, which may enhance microglial activity and contribute to persistent inflammation (Bhattacharyya et al., 2021; Zhang M. et al., 2021; Liu et al., 2022).

In recent research, specific miRNAs have emerged as critical regulators of innate responses to infections, and symbiotic bacteria have been found to downregulate the expression of miRNA-10a in dendritic cells through interactions with toll-like receptors (TLRs) and ligands (Xue et al., 2011; Sil et al., 2020; Arzua et al., 2021; Zhao et al., 2021). Another type of non-coding RNA, H19, plays a significant role in pro-inflammatory responses induced by pathogens. It achieves this through modulation of the NF-κB signaling pathway. Moreover, H19 has implications for glucose metabolism and insulin-dependent signaling, potentially linking diabetes and Alzheimer’s disease (Datta et al., 2018; Li et al., 2019).

The altered composition of the gut microbiota is a common observation and can significantly impact the progression of Alzheimer’s disease (Barandouzi et al., 2020; Romanenko et al., 2021). The complex relationship between the gut microbiota composition and specific miRNA expression in the brain is of particular interest. This bidirectional interaction has implications for anxiety and depression-related behaviors, highlighting the potential role of non-coding RNAs as mediators in the gut–brain axis. Studies suggest that disturbances in the gut microbiota can trigger processes such as apoptosis, ROS generation, and neurodegeneration (Khan et al., 2020). It is worth noting that miRNAs influenced by pathogens may play a role in neuropsychiatric disorders, further supporting the theory of the gut microbiota–ncRNA–brain axis (Rosa et al., 2022).

In summary, disturbances in the gut microbiota can lead to fluctuations in ncRNA modulation profiles, ultimately resulting in brain dysfunction (Hou et al., 2022). Table 1 provides a more detailed exploration of gut microbiome derivatives implicated in AD pathology.

**Table 1:** Gut microbiome-derived products in Alzheimer’s disease (AD) pathology.

Gut-derived products	Beneficial effect on brain functions	Detrimental effect on brain functions
BFT and BF-LPS		BFT is found to be among the most pro-inflammatory lipoglycans—BFT alters GI tract and BBB structure, integrity, and permeability (Lukiw et al., 2021)
LPS		In gut microbial dysbiosis associated with aging, the increased paracellular permeability of the gut permits LPS to escape into the bloodstream. The BBB becomes more permeable with age, allowing circulating pro-inflammatory LPS to enter the brain tissue—LPS can be used as a biomarker of AD (Hasavci and Blank, 2022)
SCFAs (e.g., butyrate)	Neurogenesis, microglial maturation, reduction of cognitive impairments	Swer et al. (2023)
GABA	Neuroprotective effect in neurodegenerative conditions	Reduced GABA levels in the brains of AD patients have been reported (Kaur et al., 2019)
Histamine		Histamine elevates nitric oxide (NO) levels and induces neuroinflammation (Zhang Y. et al., 2021)

This table provides a summary of the impact of gut microbiome-derived products on brain functions within the context of Alzheimer’s disease (AD) pathology. These products can have both beneficial and detrimental effects, highlighting the intricate relationship between gut health and brain function in AD.BFT, Bacteroides fragilis toxins; LPS, lipopolysaccharide; SCFA, short-chain fatty acids; GABA, gamma-aminobutyric acid; NO, nitric oxide; AD, Alzheimer’s disease.

### 4.1 Positive effects of microbiome changes

**Figure 3:**
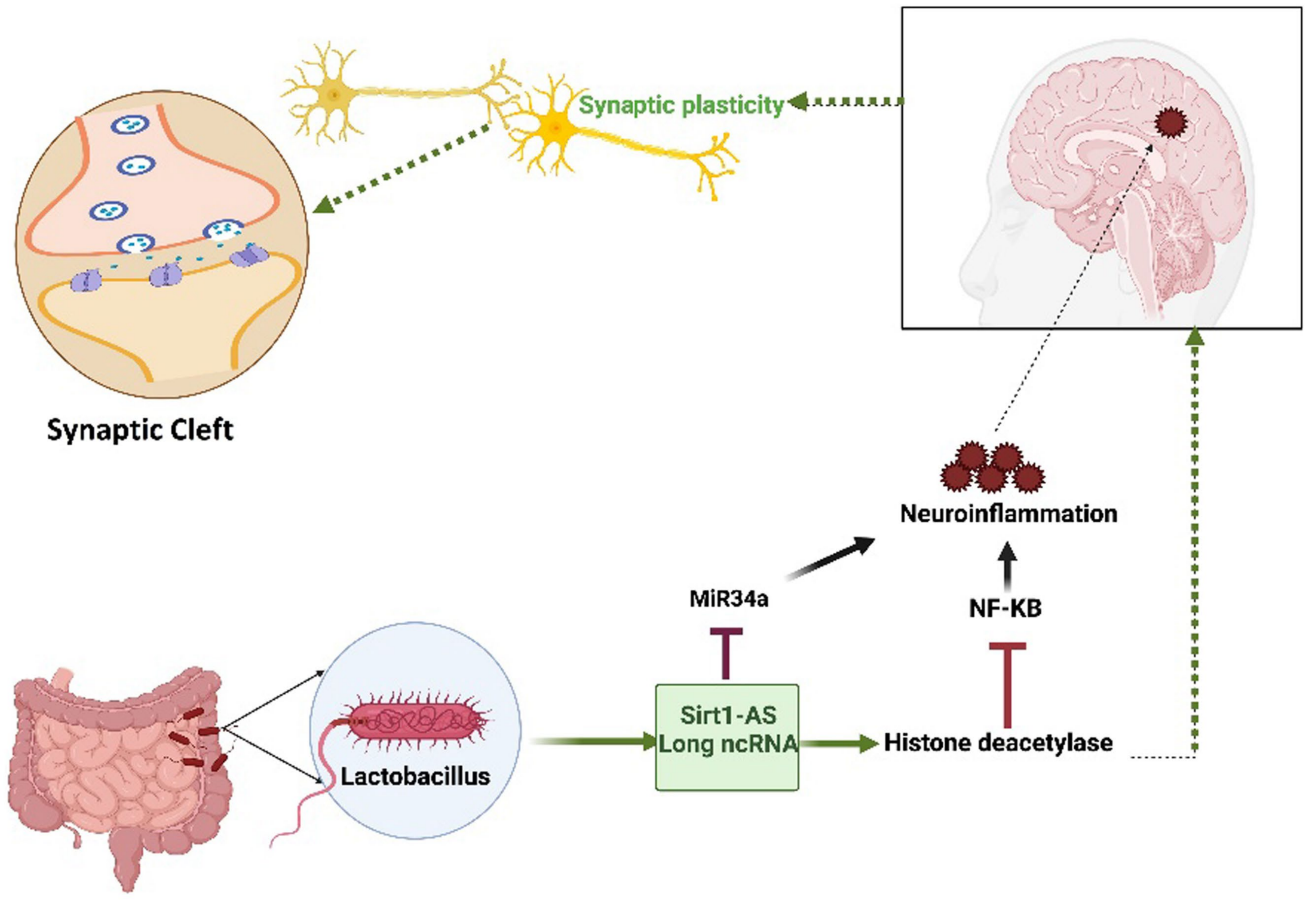
The Impact of the *Lactobacillus*-mediated regulation of Sirt1-AS, a long non-coding RNA, on human brain health. *Lactobacillus*-produced lactate plays a crucial role in orchestrating the activation of Sirt1-AS, a long non-coding RNA, effectively outcompeting miRNA-34a to protect the Sirt-1 mRNA transcript. This transcript encodes the histone deacetylase enzyme, which is known for its pivotal role in enhancing synaptic plasticity. Furthermore, this enzyme has the ability to effectively reduce neuroinflammation by inhibiting NF-KB signaling. Given its reliance on nicotinamide adenine dinucleotide (NAD^+^) and predominant localization within mitochondria, any disruption in mitochondrial function may exacerbate neurodegenerative pathologies. The figure underscores the intricate interplay between microbiota-derived molecules, RNA regulation, and neuroprotective mechanisms, offering promising therapeutic prospects.

The human gut microbiome (GM) plays a pivotal role in maintaining overall health and can significantly impact various disease states, as depicted in Figure 3. Notably, certain *Lactobacillus* strains originating from the GM have a positive impact on human brain health. Extensive research has provided compelling evidence that a metabolite called lactate, produced by the *Lactobacillus* family, contributes to the upregulation of sirtuin1 deacetylase (Sirt1) through the mediation of Sirt1 antisense (AS) lncRNA. This particular lncRNA engages in competitive interactions with miRNA-34a, ultimately rescuing the Sirt1 mRNA transcript from repression. The intricate mechanism involving lactate-induced neurotrophic factors in the hippocampus, facilitated by the Sirt1-AS lncRNA, indirectly leads to enhanced synaptic plasticity (Wang et al., 2016; Bostanciklioğlu, 2019; El Hayek et al., 2019; Ahmed et al., 2022). This connection underscores the significance of the gut–brain axis in neurodegenerative conditions and opens avenues for therapeutic interventions (Figure 3).

### 4.2 Negative effects of microbiome changes

There is growing interest in understanding how the GM might impact neurodegenerative disorders such as Alzheimer’s disease (AD), as indicated by Kaur et al. (2019), Leblhuber et al. (2021), and Hasavci and Blank (2022). The connection between the GM and AD involves a range of pathological mechanisms, including deposition of amyloid-β, hyperphosphorylation of tau, neuroinflammation, mitochondrial dysfunction, and metabolic alterations (Wang et al., 2016; Leblhuber et al., 2021; Chandra et al., 2023). Non-coding RNAs (ncRNAs) have emerged as key regulators of the host’s responses to pathogenic microbiota. However, it is crucial to underscore the importance of conducting rigorous investigations to validate the interactions between pathogens and ncRNAs within the context of the brain.

## 5 Human bacterial infections and Alzheimer’s disease

Bacterial metabolites and components, including substances such as LPS, peptidoglycan (PG), and lipoprotein, possess the ability to influence the expression of host non-coding RNAs (ncRNAs), particularly microRNAs (miRNAs), which can lead to the development of pro-inflammatory conditions that indirectly impact neurodegeneration. An example of this is miRNA-1224, which significantly increases following exposure to LPS, thereby contributing to LPS-induced inflammation by regulating tumor necrosis factor-alpha or (TNF-α) through an SP1 binding site, associated with increased levels of miRNA-1224 in AD patients, suggesting a potential link between LPS, a component of Gram-negative bacteria, and the pathogenesis of AD (Niu et al., 2011; Candido et al., 2019). Furthermore, it is noteworthy that, microbial agents often employ the NF-κB signaling pathway, mediated by miRNA-146a, as a means to promote neurodegeneration.

### 5.1 Periodontitis, nasal microbiome, and Alzheimer’s disease

Severe periodontitis can have a significant impact on tooth health and increase the risk of various diseases, including AD. Periodontitis can trigger the release of pro-inflammatory cytokines, potentially leading to increased deposition of Aβ in the brain. However, the intricate relationship between periodontitis and Alzheimer’s disease requires further research for a comprehensive understanding. In particular, *Porphyromonas gingivalis* (*P. gingivalis*), a periodontal pathogen, has been detected in the brains of AD patients and is associated with neurotoxicity. *P. gingivalis* triggers toll-like receptor (TLR-2) and TLR-4 signaling, leading to the activation of TNF-α and interleukine-1 (IL-1) through bacterial LPS and an increase in miRNA-9 expression (upregulation), ultimately alleviating inflammation (Ishida et al., 2017; Dominy et al., 2019; Bashir and Khan, 2022). LPS also induces the long non-coding RNA (lncRNA) MALAT1 in gingival fibroblasts, leading to pro-inflammatory states. Paradoxically, enhancing MALAT1 expression in AD brains has been linked to the inhibition of neuroinflammation (Gholami et al., 2020; Liu et al., 2022). Moreover, the lncRNA ANRIL, which is altered in periodontitis, is associated with AD-related neuroinflammation by binding to miRNA-125a, leading to its increased expression (Liu et al., 2022). While both MALAT1 and ANRIL are influenced by periodontitis, MALAT1 appears to contribute to neuroprotection, whereas ANRIL has neurodegenerative consequences (Liu et al., 2022). It is important to note that dysfunctions in olfaction can significantly impact mood and behavior. The miRNA-200 family plays a crucial role in olfactory neurogenesis, and there is an increasing interest in the role of the nasal microbiome in olfactory function and its potential connection to neurodegenerative disorders (Choi et al., 2008). Some bacteria in the nasal cavity may have links to the central nervous system. For instance, *Chlamydia pneumoniae*, associated with sinusitis, has been found in the brains of Alzheimer’s disease patients. Additionally, *Corynebacterium diphtheriae*, known to produce diphtheria toxin, can enter the CNS, potentially contributing to sporadic AD (Thangaleela et al., 2022a). Dysbiosis in the nasal microbiome may lead to inflammation, exacerbating neurodegeneration (Thangaleela et al., 2022a,b). Non-coding RNAs (ncRNAs), particularly miRNAs, may serve as key mediators in the nasal microbiota-brain axis. Studies have shown that elevated levels of miRNA-206 and inflammatory mediators are observed in human astrocytes exposed to LPS, and in early dementia patients, overexpression of miRNA-206 is associated with suppressed brain-derived neurotropic factor (BDNF), potentially aggravating neuropathogenesis in AD patients (Duan et al., 2015).

## 6 Human virus infection and Alzheimer’s disease

The role of viruses in neurodegenerative diseases, including AD, is garnering attention. Non-coding RNAs, particularly miRNAs, are crucial in regulating viral replication and infection outcomes. Certain infectious agents, particularly those affecting the central nervous system (CNS), are associated with Alzheimer’s disease (AD) pathology, some of which can remain latent in the CNS and reactivate with age and stress, leading to subsequent inflammation, synaptic dysfunction, and neural loss (Itzhaki et al., 2016).

### 6.1 Human immunodeficiency viruses

**Figure 4:**
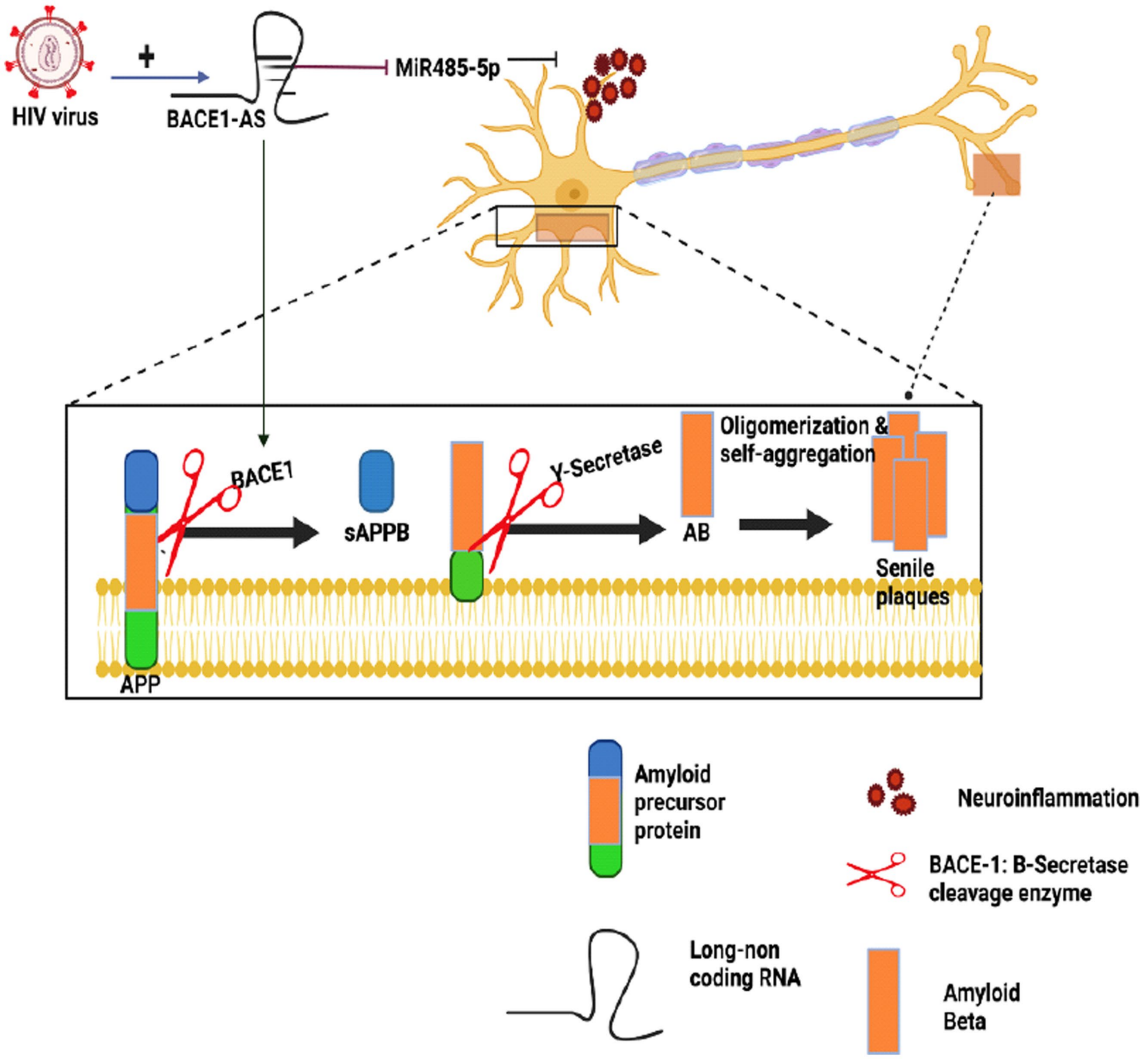
The impact of HIV on Alzheimer’s amyloidogenic pathway. This figure provides an overview of how HIV infection can influence Alzheimer’s disease (AD) mechanisms, particularly the amyloidogenic pathway. BACE1-AS acts as a competing endogenous RNA (ceRNA), sequestering miRNA-485-5p, which in turn, enhances BACE1 mRNA expression, ultimately contributing to Aβ generation in AD. Studies on CNS lentiviruses have revealed their ability to induce the expression of miRNA-31, which reduces Aβ deposition in brain regions such as the subiculum and hippocampus, and this suggests a potential role for non-coding RNAs (ncRNAs) in the pathology of AD. However, due to the intricate and sometimes contradictory effects of diverse lentiviruses on AD, further comprehensive investigations remain essential to fully understand this complex interaction.

In a study conducted in 2020, HIV-infected patients exhibited elevated BACE1 enzyme expression and increased production of Aβ (Moloney et al., 2017; Zhao and Lukiw, 2018a) in human astrocytes exposed to Tat, an early gene product of HIV-1 continually secreted from latently infected cells. This mechanism involved the upregulation of hypoxia-inducible factors-1-alfa (HIF-1α), leading to the formation of the lncRNA BACE1-antisense (AS/BACE1) RNA complex. Subsequently, the complex increased BACE1 enzyme activity (Sil et al., 2020). Figure 4 illustrates how BACE1-AS functions as a competing endogenous RNA (ceRNA), sequestering miRNA-485-5p and relieving its inhibitory effect on BACE1 mRNA, ultimately resulting in increased BACE1 mRNA expression (Khan et al., 2020; Canseco-Rodriguez et al., 2022; Ryu et al., 2023). Research on lentiviruses in the CNS revealed that these viruses can induce the expression of miRNA-31, which significantly reduces Aβ deposition in the subiculum and hippocampus. This finding suggests a potential role for non-coding RNAs (ncRNAs) in Alzheimer’s disease (AD) neuropathology (Barros-Viegas et al., 2020). Contradictory effects of different lentiviruses on Alzheimer’s disease (AD) call for further comprehensive investigations.

### 6.2 Hepatitis B viruses

In patients infected with hepatitis B virus (HBV), a study observed a significant induction of miRNA-125b-5p in serum, which was associated with the persistence of the HBV infection. Another study revealed that the upregulation of miRNA-125b is linked to an increase in tau protein hyperphosphorylation in AD, which contributes to the progression of the disease (Singh et al., 2018).

### 6.3 Severe acute respiratory coronavirus 2

In the context of viral infections, it has been reported that during Severe acute respiratory coronavirus 2 (SARS-CoV2) infection, miRNA-200c-3p disrupts the regulation of the angiotensin-converting enzyme (ACE), indirectly inhibiting viral infection (Sodagar et al., 2022). Furthermore, a study in 2022 suggested that miR-200c suppression increases tau hyperphosphorylation by targeting 14-3-3y at the early-stage AD in a mouse model (5xFAD; Park et al., 2022). These findings highlight the bidirectional impact of ncRNAs on microbes in disease development or suppression (Abu-Izneid et al., 2021).

### 6.4 Herpes simplex viruses

Herpes Simplex Virus type 1 (HSV-1) infection is recognized as a contributing factor to the progression of Alzheimer’s disease (AD). It is found in the latent form within the brains of many older individuals, leading to neural and glial cell damage and immune dysfunction. HSV-1 can enter the CNS without clinical symptoms, and its reactivation can accelerate the pathogenesis of AD (Mielcarska et al., 2021). HSV-1 expresses its own miRNAs abundantly during latency and has the capacity to manipulate host miRNAs to facilitate either replication or latency (Cokarić Brdovčak et al., 2018). The co-evolution of the host and virus enables HSV-1 to hijack host cellular processes for its growth and survival. Non-coding RNAs, particularly miRNAs, play a crucial role in viral pathogenesis by balancing latency and reactivation in response to various stimuli. For example, cellular miRNA-101 targets mitochondrial ATP synthase, reducing the lytic replication of HSV-1 and promoting latency (Zheng et al., 2011). In contrast, miRNA-23a supports viral replication by inhibiting the host’s antiviral innate immune response (Ru et al., 2014). Another study revealed that HSV-1 infection of human neuronal glial cells induced miRNA-146a, which targets key components of the arachidonic acid pathway and fumarate hydratase (FH), enabling HSV-1 to evade the complement system, exacerbating neuropathological changes in AD (Hill et al., 2009; Zhao and Lukiw, 2018a; Slota and Booth, 2019). HSV-1 infection has also been associated with tau hyperphosphorylation, contributing to neurotoxicity in AD brains (Alvarez et al., 2012). In summary, viral and cellular miRNAs and their interplay appear to play a significant role in viral latency and reactivation, thereby influencing virus replication and pathogenesis (Bernier and Sagan, 2018). Consequently, miRNAs are strongly associated with viral-related pathological events (Piedade and Azevedo-Pereira, 2016).

### 6.5 Human cytomegalovirus

Human cytomegalovirus (HCMV) causes severe disease, especially in individuals with compromised immune systems. It is linked to the early onset of systemic sclerosis and has the capability to enter the CNS, causing inflammation and histopathological changes in various brain cells. CMV-induced inflammation mediated by the host can exacerbate immunopathology and neuropathological symptoms (Krstanović et al., 2021). Studies have reported several AD pathological hallmarks associated with neuro-invasive HCMV, including the accumulation of amyloid-beta (Aβ), astrogliosis, neuroinflammation, and neural damage. *In vitro* experiments have shown that HCMV can induce Aβ production and astrocyte reactivity, resembling AD pathological features (Adelman et al., 2023). HCMV encodes its own microRNAs (miRNAs) that target viral genes and host cellular ones, including immunomodulators. Additionally, it can modify cellular miRNAs to facilitate their replication (Dhuruvasan et al., 2011). HCMV can manipulate various cellular mechanisms to its advantage, affecting host metabolic pathways and cell apoptosis (Das et al., 2007; Leng et al., 2017). Notably, downregulation of the miRNA-199a/214 cluster has been observed in HCMV-infected cells (Dhuruvasan et al., 2011). In a study from 2020, miRNA-199a was found to decrease neuritin expression, contributing to AD progression in mouse models. Researchers noted significant miRNA-199a expression at the early stages of AD, suggesting its potential as a biomarker for early disease diagnosis (Song et al., 2020).

## 7 Conclusion

The early diagnosis of Alzheimer’s disease is critical for delaying or mitigating its progression. Non-coding RNAs (ncRNAs) present in blood circulation and the cerebrospinal fluid hold promise as reliable diagnostic biomarkers due to their stability and ease of detection. Moreover, ncRNAs have therapeutic potential, particularly in the preclinical stages of the disease. However, the development of effective drugs remains challenging due to a limited understanding of AD pathogenesis, and current drugs only offer symptomatic relief. Therefore, in-depth research into the causes of AD is essential. The regulatory role of ncRNAs in various human diseases, including the potential influence of microorganisms within the human body on host ncRNAs, presents a promising avenue for investigation. Studying the molecular basis of AD pathology, especially the mechanisms involving ncRNAs, is crucial for advancing rational treatment approaches. Accumulated amyloid-beta (Aβ), a hallmark of AD, can trigger microglia activation and subsequent neuroinflammation, with many ncRNAs, including miRNAs, playing key roles as modulators of neuroinflammation. Some ncRNAs also regulate enzymes involved in Aβ processing, highlighting their potential impact on AD pathology. Dysregulations of these ncRNAs, influenced by external factors such as viruses and bacteria, can significantly alter disease trajectories. Recent research has shed light on the role of gut and nasal microbiota in neurodegenerative diseases, emphasizing the role of ncRNAs in modulating immune responses and inflammation. Investigating the profiles of ncRNAs responsible for controlling pro-inflammatory genes associated with AD is essential for effective treatment or early disease diagnosis. Perturbations in neuroinflammation-related ncRNAs within various microbiome communities may lead to the dysregulation of specific signal transduction pathways, contributing to disease development. To improve treatment and diagnosis methodologies for Alzheimer’s disease, further research is needed to explore the roles of ncRNAs in regulating genes involved in the progression of the disease.

## Author contributions

HM-P: Visualization, Writing – original draft, Conceptualization. MS: Supervision, Writing – review & editing, Conceptualization, Project administration. FJ: Data curation, Supervision, Writing – review & editing. HN: Data curation, Writing – review & editing. FM-G: Writing – review & editing, Supervision.
